# A Novel Role for Extracellular Vesicles in Cytopathology and New Therapeutic Strategies

**DOI:** 10.1155/2019/7137613

**Published:** 2019-11-11

**Authors:** Shi-Cong Tao, Shang-Chun Guo, Johnny Collett

**Affiliations:** ^1^Department of Orthopaedic Surgery, Shanghai Jiao Tong University Affiliated Sixth People's Hospital, Shanghai, China; ^2^Institute of Microsurgery on Extremities, Shanghai Jiao Tong University Affiliated Sixth People's Hospital, Shanghai, China; ^3^Faculty of Health and Life Sciences, Oxford Brookes University, Oxford, UK

Extracellular vesicles (EVs) are a newly discovered way by which cells communicate with their neighbouring cells. EVs are lipid nanovesicles that enclose proteins (membrane proteins and internal proteins) and nucleic acids (miRNA, mRNA, lncRNA, and circRNA). EVs include exosomes having a diameter of 30–100 nm (called “small EVs” (sEVs), according to the “Minimal Information for Studies of Extracellular Vesicles 2018,” or “MISEV2018,” guidelines) and microvesicles having a diameter of 100–1,000 nm (called “large/medium EVs” (l/mEVs), according to the MISEV2018 guidelines). These sEVs and l/mEVs are generated and released from most cell types and, hence, are found in most biological fluids. In particular, sEVs have increasingly attracted attention for their role in physiology and pathology, as well as their possible use as diagnostic and therapeutic tools.

This special issue compiles a series of 5 original contributions and 6 comprehensive reviews that, while not being a complete representation of the field, are an important collection of multifaceted works; we have the pleasure of sharing this knowledge with our readers. These articles cover the relevant aspects of physiology, pathogenesis, diagnoses, and therapies that involve EVs.

X. Wu et al. reported a study that evaluated whether the miRNAs enclosed in circulating sEVs have the predictive values in patients at risk of developing acute respiratory distress syndrome (ARDS) from severe community-acquired pneumonia (SCAP). They found a subset of sEVs carrying miRNAs, including miR-146a, miR-27a, miR-126, and miR-155, in ARDS group samples that exhibited significantly elevated levels than those in non-ARDS group samples. The combined expression of miR-126, miR-27a, miR-146a, and miR-155 predicted ARDS with an area under the curve of 0.909 (95% CI 0.815–1). Only miR-126 had the potential to predict 28-day mortality (OR = 1.002, *P*=0.024) with its median value classifier. The authors suggest that altered levels of circulating sEVs carrying miRNAs may be a useful biological confirmation of ARDS in patients with SCAP.

C. Dong et al. described the potential roles of circulating sEVs carrying miRNAs during mesenchymal stem cell (MSC) senescence, which is crucial in the development and progression of systemic lupus erythematosus (SLE). The authors found that sEVs derived from the SLE serum increased the proportions of SA-*β*-gal positive cells, disorganized cytoskeleton structures, and reduced growth rates in MSCs. In SLE patients, miR-146a declined significantly in the level of serum sEVs, compared with healthy controls. This reduction in miR-146a levels could be involved in a novel mechanism of MSC senescence in SLE patients through the targeting of TNF receptor associated factor 6 (TRAF6)/nuclear factor kappa-B (NF-*κ*B) signalling.

J. Wang et al. used high-throughput sequencing to explore the miRNA expression profiles of sEVs in the peripheral blood of kidney recipients having delayed graft function (DGF). They identified 52 known and 5 conserved sEVs containing miRNAs specifically expressed in recipients having DGF. Three coexpressed miRNAs, hsa-miR-33a-5p_R-1, hsa-miR-98-5p, and hsa-miR-151a-5p, were found to be significantly upregulated in kidney recipients with DGF. Moreover, hsa-miR-151a-5p was positively correlated with the first-week serum creatinine (CR), blood urea nitrogen (BUN), and uric acid (UA) levels in the kidney recipients, after transplantation. In addition, they also analysed functions and signalling pathways of the 3 upregulated miRNA target genes to uncover a putative mechanism of how these miRNA-carrying sEVs functioned during DGF.

J. Ding et al. investigated whether sEVs originated from bone marrow-derived mesenchymal stem cells (BMSCs) preconditioned by deferoxamine (called DFO-sEVs), and explored the mechanisms underlying their superior proangiogenic properties during wound repair. They reported that, in cell-free therapies, DFO-sEVs activate the phosphoinositide 3-kinase (PI3K)/AKT signalling pathway via miR-126-mediated phosphatase and tensin homolog (PTEN) downregulation to enhance healing and angiogenesis in diabetic wounds.

K. Saito et al. are the first to report that second-generation antihistamines, including cetirizine, fexofenadine, azelastine, and terfenadine, exert suppressive effects on Kv1.3-channels in lymphocytes. The efficacy of these drugs may be related to their immunomodulatory mechanisms that reduce inflammatory cytokine synthesis. As a matter of fact, EVs play an important role in inflammatory cells. In a recent study, lymphocytes were found to release EVs which may contribute to type 1 diabetes development. Meanwhile, ion regulatory channels were found to be closely related to EVs. Thus, their findings may suggest a new breakthrough point in extracellular vesicles and new tools for studying them.

R. Dong et al. reviewed the current understanding of sEVs derived from mesenchymal stem cells (MSCs) related to peripheral nerve repair and provide insights on the development of a cell-free MSC therapeutic strategy for peripheral nerve injury (PNI).

J. Lu et al. reviewed the possible relationships between risk factors for bone nonunion and EVs and then discussed the roles of EVs in bone metabolism and regeneration. More and more literature points out that EVs are likely to be the way cells (including osteoclasts, osteoblasts, osteocytes, endothelial cells, and MSCs) communicate with each other in the process of bone healing via the exchange of bioactive substances (such as proteins and nucleic acids). Beyond that, EVs can be used to deliver functional RNAs and mediate cell-to-cell communication, suggesting that EVs may repair bone defects by regulating cells and cytokines involved in bone metabolism.

W. Zhao et al. reviewed the roles of emerging sEV-carried long noncoding RNAs (lncRNAs) in cancer. In addition, the biogenesis of sEVs, the functions of lncRNAs, and the mechanisms of lncRNAs in sEV-mediated cell-cell communication were also summarized.

W. Tian et al. reviewed the latest literature to summarize the action mechanisms and experimental studies of sEVs in cancer metastasis. Studies have shown that sEVs play a vital role in cancer metastasis in forming the premetastatic niche, influencing tumor cells and microenvironment, and determining specific organotropic metastasis. They also raised open questions about the influence of sEVs on metastasis: “(a) Are exosomes still playing a role during tumor latency or after primary tumor resection? (b) What causes the difference of exosomal biomarker levels in serum and plasma? (c) What are the mechanisms governing the specific exosomal cargo targeting between tumor and recipient cells which contribute to inconsistent expression of exosomal inclusions in blood and tissue?”

Z. Zhang et al. summarized recent findings on the function of noncoding RNAs (ncRNAs) and then studied their interaction with premetastatic niche formation, which highlighted the potential of using EVs carrying ncRNAs for cancer diagnosis and therapeutic strategies against it.

S. Li et al. reviewed the role of sEVs in hepatitis B virus (HBV) infection and discussed the following: (1) direct participation of sEVs in HBV replication; (2) modulation of immune responses by sEVs during HBV infections; (3) sEVs carrying RNAs and proteins, which could be novel biomarkers for the diagnosis of HBV infections; and (4) sEVs designed as vaccines against HBV.

